# HybridGNN: a graph neural network approach for human miRNA–disease association prediction

**DOI:** 10.1093/bioinformatics/btag171

**Published:** 2026-04-04

**Authors:** Basharat Ahmad, Muhammad Hammad Musaddiq, Sebu Aboma Temesgen, Grace-Mercure Bakanina Kissanga, Huma Fida, Hao Lin, Ye-Chen Qi

**Affiliations:** School of Life Science and Technology, University of Electronic Science and Technology of China, Chengdu 611731, China; School of Information and Communication Engineering, University of Electronic Science and Technology of China, Chengdu 611731, China; School of Life Science and Technology, University of Electronic Science and Technology of China, Chengdu 611731, China; School of Life Science and Technology, University of Electronic Science and Technology of China, Chengdu 611731, China; School of Life Science and Technology, University of Electronic Science and Technology of China, Chengdu 611731, China; School of Life Science and Technology, University of Electronic Science and Technology of China, Chengdu 611731, China; School of Life Science and Technology, University of Electronic Science and Technology of China, Chengdu 611731, China

## Abstract

**Motivation:**

MicroRNAs (miRNAs) are small non-coding RNAs, typically 18–24 nucleotides in length, that play a pivotal role in RNA silencing and the post-transcriptional regulation of gene expression by targeting messenger RNAs (mRNAs). Dysregulation of these miRNAs has consistently been implicated in the onset and progression of a variety of complex human diseases.

**Results:**

In this study, we propose a novel *HybridGNN* model that integrates a Graph Convolutional Network (GCN), a Graph Attention Network (GAT), and Matrix Decomposition with Matrix Factorization (MDMF) to predict potential miRNA–disease associations (MDAs). We incorporate five types of similarity in which three are derived from miRNAs and two are derived from diseases, to comprehensively explore and optimize multi-source feature information. The complementary interactions among these modules also help to mitigate the oversmoothing problem. The model utilizes neighboring nodes in a heterogeneous network to generate node embeddings via a message-passing mechanism. To improve computational efficiency, we employ a mini-batch gradient descent approach that partitions the graph into smaller sub-graphs, thereby enhancing the model’s accuracy, speed, and scalability. As a result of these advanced techniques, HybridGNN achieved an area under the receiver operating characteristic curve (AUC-ROC) of 0.9715 using a dot-product classifier, outperforming several existing methods and underscoring its potential as a robust and accurate tool for predicting MDAs.

**Availability:**

Code and data are freely available at https://github.com/mbasharatahmad/HybridGNN-miRNA-disease/

## 1 Introduction

The human genome comprises of vast genetic material but only 1.5% of genes are responsible for coding proteins and majority are transcribed into non-coding RNAs (ncRNAs). These ncRNAs play pivotal roles in fundamental biological process, including tissue differentiation and regulation of cell cycle (George *et al*. 2024). These RNAs are commonly classified based on the transcript length, those shorter than 200 nucleotides are referred to as small ncRNAs, whereas transcripts longer than 200 nucleotides are termed long non-coding RNAs (lncRNAs) (Liu *et al*. 2024b). Among small ncRNAs, microRNAs (miRNAs) represent a well-known characterized class of single-stranded, evolutionarily conserved molecules, typically comprising 18–24 nucleotides (Liu *et al*. 2024a, Saw and Song 2025). miRNAs regulate gene expression at the post-transcriptional level by binding to target messenger RNAs (mRNAs), resulting in either mRNA degrading or translation inhibition (Wei *et al*. 2021, Bakhti and Latifi-Navid 2022, Lin *et al*. 2025, Luo *et al*. 2025). Numerous miRNAs are associated with various diseases including, miR-143/145 family is linked to hypertension, miRlet‐7 and miR‐103/107 regulate of glucose metabolism; miR-29 is involved in diabetes; and miR‐17–92 cluster, contribute to cancers development (Chistiakov *et al*. 2016, Wojciechowska *et al*. 2017, Saliminejad *et al*. 2019).

Identification of disease-associated miRNAs can facilitate pathological classification of diseases and contribute to the development of individualized treatment strategies (Tang *et al*. 2018, Mamatkarimov *et al*. 2025, Popa *et al*. 2025, Qi *et al*. 2025, Wang *et al*. 2025b, Xie *et al*. 2025). Experimental approaches can accurately identify ncRNA–disease associations but are constrained by high cost and time-consumption (Lv *et al*. 2024, Qiao *et al*. 2024, Sheng *et al*. 2024). Recently significant advances have been made in computational methods for identifying potential miRNA–disease association (MDAs) (Chen *et al*. 2025b) based on network biology, mainly including similarity-based and machine learning-based approaches (Perez-Iratxeta *et al*. 2005, Ai *et al*. 2024, Wang *et al*. 2023, Yizheng Wang *et al*. 2024). Computational models such as, DeepMDA which is proposed by Fu *et al*. (2017) that predict MDAs by constructing heterogeneous network and extracting miRNA and disease features using the Gaussian Interaction Profile Kernel (GIPK) similarity (Fu and Peng 2017). Similarly, Ha and Park (2021) introduced (MLMD) that predict MDAs and capture miRNA-miRNA and disease–disease association. In 2018, Chen *et al*. proposed IMCMDA which uses inductive matrix completion to predict MDAs by integrating Directed Acyclic Graph (DAG) based disease similarity and GIPK based miRNA similarity (Li *et al*. 2023). In the subsequent study, the authors employed bipartite recommendation and a rating integrated bipartite network projection method to predict MDAs (Chen *et al*. 2018b).

Furthermore, various approaches based on MDAs similarity have been explored (Zeng *et al*. 2016). A novel computational method was proposed that integrates microRNA-based similarity inference, phenotype-based similarity inference and network-consistency-based inference for predicting MDAs (Wang *et al*. 2010). The researchers utilized a DeepWalk-based graph embedding method (DWMDA) to predict MDAs by extracting low dimensional features from miRNAs and disease network (Ha 2025). Additionally, a deep neural network was employed to identify MDAs, while MNEMDA, applied meta-path-based network embedding to extract features from heterogeneous network (Wang *et al*. 2025a). Recently, Chen *et al*. (2025), proposed a hybrid framework combining principal neighborhood aggregation and graph attention networks, to capture complex node feature and improve MDAs prediction (Chen *et al*. 2025a).

**Figure 1:**
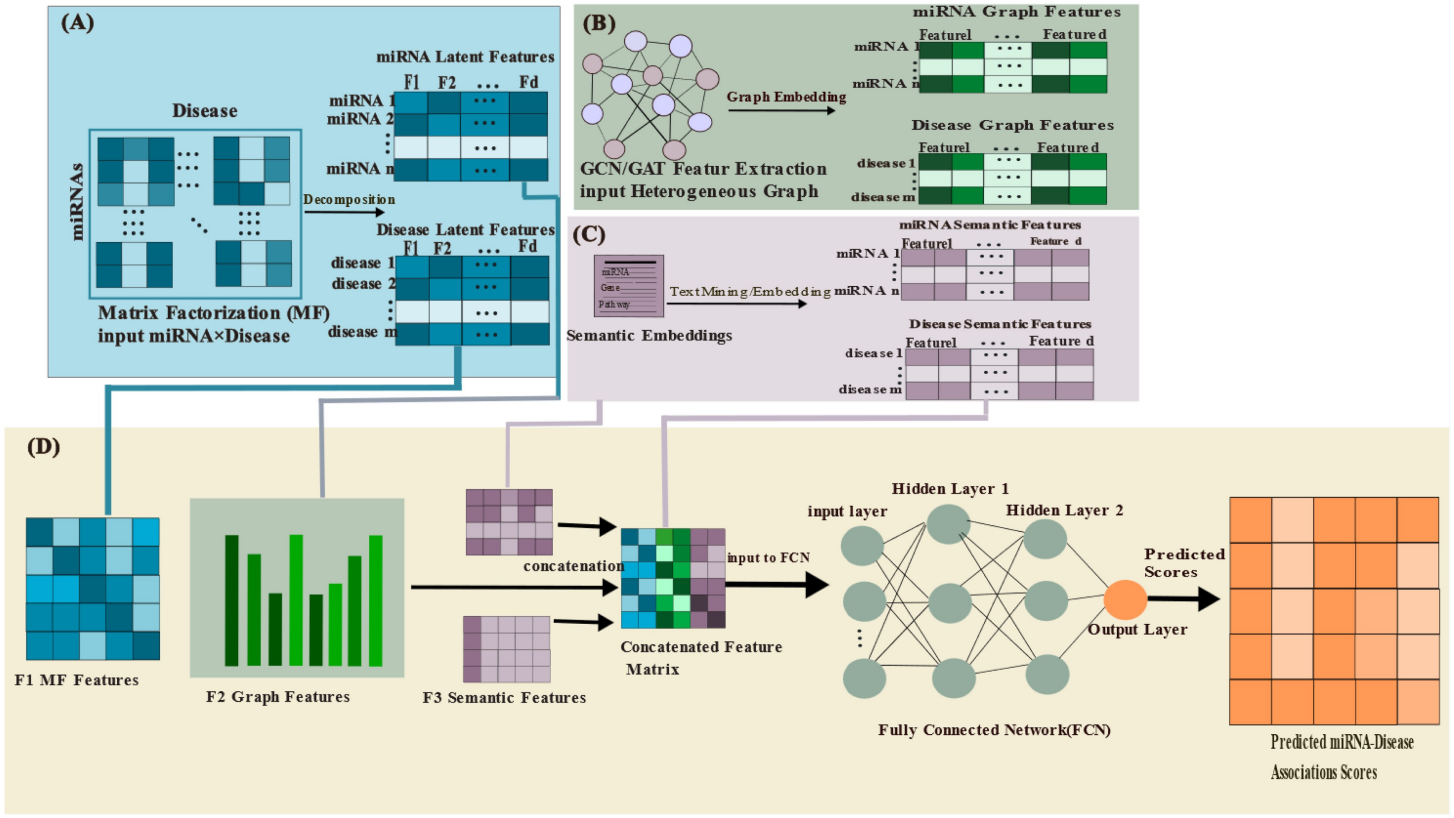
Architecture of the proposed HybridGNN model for predicting miRNA-disease associations. (A) Data preparation, including the construction of miRNA-disease association matrices and the integration into a heterogeneous network. (B) Feature extraction using MDMF, GCN, and GAT modules (C) feature representation and (D) multimodal fusion and prediction of miRNA-disease associations through a fully connected network.

To address the aforementioned limitations, we introduced a novel HybridGNN integrating MDMF and GCN-GAT, for MDAs prediction. The heterogeneous network was constructed based on miRNA, disease and their association using five similarity network and fused adjacency matrices. Three modules were used to extract sequence and topological feature of miRNA-disease, while a fully connected layer was employed to fuse the extracted feature and prevent overfitting and generate the final prediction. The complete HybridGNN model process was depicted in Fig. 1.

## 2 Methods

### 2.1 Dataset collection and processing

The data set was obtained from Human microRNA disease database (HMDD v4.0) it comprises of curated 53 531 miRNA–disease association (MDAs). After removal of duplicate entry, 728 miRNAs and 175 diseases, encompassing 18 738 known miRNA–disease associations were considered. Duplicate miRNA–disease pairs were systematically removed to construct a non-redundant dataset, in which each association is represented only once. The preprocessing step ensures data consistency and provides a reliable foundation for training and evaluation of the HybridGNN model (Cui *et al*. 2024). Additionally, to further assess the generalization ability of the proposed model we collected the IMCMDA benchmark dataset consisting of 5430 MDA introduced by Chen *et al*. (2018a).

### 2.2 miRNA association

miRNAs association play crucial role genes regulation and cellular homeostasis and their disruption can lead to various diseases therefore, computational modeling of MDA is essential, in this study the miRNA similarity matrix was constructed by averaging sequence, GIPK and functional similarities. Accordingly, the sequence similarity matrix ${\mathrm{nS}}^{\mathrm{seq}}\;$was calculated using the Equation (1) (Sun *et al*. 2022).


(1) $${nS}^{\mathrm{seq}}=\;\frac{〈s_{i}s_{j}〉}{\|\;s_{i}\;{\|}^{2}\;+\;\|\;s_{j}\;{\|}^{2}\;-\;〈s_{i}s_{j}〉}$$

where $〈s_{i}s_{j}〉$ holds the dot product values of miRNAs 4-mers sequence, vector of miRNA *s_i_* and *s_j_* which reflects the Eucliden distance between the two miRNAs. Similarly, $\|\;s_{i}\;\|\;=\sqrt{〈s_{i}s_{i}〉}$. It exhibits the Eucliden distance between the two miRNAs.


(2) $$〈s_{i}s_{j}〉=\sum_{k=1}^{n}s_{i,k\;}\times s_{j,k}$$

Where *k* represents the index of feature (from 1 to *n*), where n is the total number of features used to represent each miRNA. *s_i,k_* represents the *k*-th feature value of miRNA *i*, and *s_j,k_* represents the feature value of miRNA *j*.

### 2.3 miRNA–disease association

The adjacency matrix is a 2D array in which row indicate miRNAs and columns represents diseases. The matrix element *A* (*i*, *j*) indicates whether an association exists between the *i*-th miRNA and *j*-th disease, a value of 1 denotes a confirmed association, while 0 indicates an unknown association.


(3) $$A=\left[\begin{array}{c} a_{1,1}\\ a_{2,1}\\ \vdots \\ a_{m,1} \end{array}\begin{array}{c} \;a_{1,2}\\ \;a_{2,2}\\ \;\vdots \\ \;\;a_{m,2\;} \end{array}\;\begin{array}{c} a_{1,n}\\ a_{2,n}\\ \vdots \\ \cdots a_{m,n} \end{array}\right]$$

GIPK is used to capture similarity between miRNAs based on their disease association patterns. GIPK is calculated based on the following Equation (4) (Sun *et al*. 2024).


(4) $${nS}^{\mathrm{GIPK}}(n_{i},n_{j})=\exp({-\lambda }_{n}|A(n_{i,})-A(n_{j,}){|}^{2})$$

where *A* ($n_{i},)$and *A* ($n_{j,}$) represent the *i*-th and *j*-th row vector of the adjacency matrix *A* and $\lambda_{n}$ is the Kernel coefficient, which is specified in Equation (5).


(5) $$\lambda_{n}=\frac{1}{\frac{1}{N}\sum_{l=1}^{N}{\;|A(n_{l},)|}^{2}}$$

where *N* is the total miRNA number and *A* ($n_{l},$) is the *l*-th row vector of the adjacency matrix *A*.

We used Wang *et al*. MISIM (microRNA Functional Similarity) method (Wang *et al*. 2007) to calculate miRNA functional similarity based on their shared disease association.


(6) $${nS}^{\mathrm{fun}\;}(n_{1}{,\;n}_{2})=\frac{\sum_{i=1}^{p}\;S({\mathrm{de}}_{1i,}\;{\mathrm{DE}}_{2})+\;\sum_{j=1}^{q}\;S({\mathrm{de}}_{2j,\;}{\mathrm{DE}}_{1})}{p+q}$$

In the equation, *p* and *q* denotes number of diseases linked to miRNAs $n_{1}$ and miRNA $n_{2}$ respectively, and *S* (de, DE) measure the semantic similarity between a given disease de and the set of disease DE.


(7) $$S(\mathrm{de},\;\mathrm{DE})=\underset{1\leq i\leq j}{\max}\;(S({\mathrm{de}}_{i},\;{\mathrm{de}}_{j}))$$

*S* (${\mathrm{de}}_{i}$, ${\mathrm{de}}_{j}$) represent semantic similarity between disease i and j.

The integrated miRNA similarity is calculated as the average of sequence, GIPK and functional similarity as shown in Equation (8).


(8) $$nS=\frac{{nS}^{\mathrm{seq}}+{nS}^{\mathrm{GIPK}}+{nS}^{\mathrm{fun}}}{3}$$

### 2.4 Disease association

Diseases associations were obtained by averaging DAG-based semantic similarity and GIPK similarity derived from disease association matrices. In the DAG based semantic similarity (Nilsson *et al*. 2021), diseases sharing common ancestor are considered more closely related as shown in Equation (9).


(9) $${\mathrm{DS}}^{\mathrm{sem}}(d_{i,}\;d_{j})=\;\frac{\sum_{t\in T_{i}\;\cap T_{j}}\left(S_{d_{i}}(t)\;+S_{d_{j}}(t)\right)}{\sum_{t\in T_{i}}S_{d_{i}}(t)+\sum_{t\in T_{j}}S_{d_{j}}(t)}$$

*T_i_* and *T_j_* denote disease set for *d_i_* and *d_j_*, sharing ancestor disease in DAG structure. $S_{d_{i}}(t)$ indicates the semantic contribution of ancestor *t* to disease *d_i_*, computed in following Equation (10).


(10) {Sdk(t)=1, if dk=djSdk(t)=1, if dk=djotherwise

Where *S* denotes the semantic score of disease, $d_{k}$ denotes specific disease node under the consideration, and *t* is the node in the DAG, $d_{j}$ represents the target disease node for which the semantic score is calculated, ť refers to a child node of *t*, the max function selects the highest weighted child score and ∂ is a decay factor set to 0.5.

The GIPK similarity among various diseases is calculated based on the following Equation (11).


(11) $${\mathrm{DS}}^{\mathrm{GIPK}}(d_{i,}d_{j})=\exp(-\lambda_{d}|A(,\;d_{i})-A(,\;d_{j}){|}^{2})$$

where *A* (, $d_{i}$) and *A* (, $d_{j}$) hold the *i*-th and *j*-th column vectors of the adjacency matrix *A* and $\lambda_{d}\;$represents the Kernel coefficient which is specified in Equation (12).


(12) $$\lambda_{d}=\frac{1}{\frac{1}{D}\;\sum_{l=1}^{D}|A(,\;d_{l}){|}^{2}}$$

where *D* represents the total number of diseases and *A* (, $d_{l}$) represents the *l*-th column vector of adjacency matrix *A*. Then, average association among diseases is calculated as shown in:


(13) $$\mathrm{DS}=\frac{{\mathrm{DS}}^{\mathrm{sem}}+\;{\mathrm{DS}}^{\mathrm{GIPK}}}{2}$$

### 2.5 Constructing of miRNA-disease heterogeneous network

A heterogeneous graph comprises of multiple nodes and edges, each representing unstructured data sources. Following the work of Chen *et al*. (2022), a heterogeneous graph is defined as:


(14) $$G=(V,\;E,\;D,\;R)=[\begin{array}{cc} SS & A^{T}\\ A & DS \end{array}]$$

where *V* and *E* represent the sets of nodes and edges, the *D* and *R* signify node and edge types. The heterogeneous network integrates SS (miRNA similarity), *A* (miRNAs–disease association), $A^{T}$(representing reverse association), and DS (diseases similarity). following Chen *et al*. (2022), a network embedding function *f: V→ X ∈ R^|V|×b^* (where *b ≪ |V|*), maps node into a low-dimensional space effectively modeling both known and unknown miRNA-disease interactions, in Equation (14).

This hypergraph learning, capture high order relations, it may produce inaccuracies when applied to limited or noisy heterogeneous data (Kesimoglu and Bozdag 2024). To address this, we designed a hybrid multimodule framework combining Matrix Decomposition and Matrix Factorization (MDMF), Graph Convolutional Networks (GCN), and Graph Attention Networks (GAT) module.

### 2.6 Feature extraction

#### 2.6.1 Matrix factorization-based feature extraction (MDMF)

We employed matrix decomposition and matrix factorization (MDMF) approach to extract latent representation from the MDAs matrix *A*. First, matrix factorization approximates the original matrix as:


(15) A ≈ W H

where, *W* represents latent feature matrix of miRNAs and *H* indicates latent feature matrix of diseases. This step projects the sparse and high-dimensional association matrix into a shared low-dimensional latent space. Based on this low-rank representation, the matrix is further expressed as:


(16) A = B C

where, *B* denotes basic components matrix, and *C* represents the reconstruction coefficients matrix. The matrices *B* and *C* are derived from and structured upon the latent space learned through *W* and *H*. Thus, the second formulation is a refined reconstruction of the first factorization, reorganizing the learned latent features into basis components and coefficients to enhance pattern extraction and dimensionality reduction. The resulting refined embeddings (Feature1, F1) are then used as input to the HybridGNN model for downstream prediction.

#### 2.6.2 Association network representation module based on GCN

We constructed a three layer of GCN-based module, that takes miRN-disease heterogeneous association network as input and using integrated miRNA and disease similarity matrices as features, with message passing as shown in Equation (17).


(17) $$H_{l+1}=\;\sigma ({\tilde{D\;}}^{-(1/2)}{\tilde{\;A}}_{h}{\tilde{D}}^{-(1/2)}\;H_{l}W_{l})$$

here $\tilde{A_{h}}$ = $A_{h}+I_{N}$, where $A_{h}$ is the adjacency matrix and $I_{N}$ is identity matrix. $\tilde{D\;}$denotes the degree matrix with diagonal element. $\tilde{D_{ii}}=\sum_{j}\tilde{A_{h_{ij}}}$. $H_{l}$ is feature matrix at the *l*-th layer, $W_{l}$ the trainable weight matrix and *σ* (·) displays the Relu activation function. This module produces the feature matrix *F*2.

#### 2.6.3 Topological structure representation module based on GAT

To dynamically weights nodes importance, a 3-layer GAT architecture with a single attention head per layer were used (Momanyi *et al*. 2024a, 2024b, Dai *et al*. 2025).


(18) $$h_{i}=\sigma (\sum_{{j\in N}_{i}}\alpha_{i,\;j}\;W_{l}H_{j})$$

Where $h_{i}$ represents the feature vector of node *i* at the *l*-th layer, $N_{i}$ denotes the set of neighbor’s nodes connected to node i in heterogeneous network, $W_{l}$ is the trainable weight matrix at *l*-th layer attention coefficients $\alpha_{i,\;j}$ is the normalized attention score as shown in Equation (19).


(19) $$\alpha_{i,j}=\mathrm{softmax}\;(e_{i,\;j})=\frac{\exp(e_{i,\;j})}{\sum_{{k\in N}_{i}}\exp(e_{i,\;k})}$$

Where $e_{i,\;j}\;$denotes self-attention score, this matrix captures higher order relational dependencies for improved link prediction, and outputs the feature matrix *F*3.

### 2.7 Predicting miRNA-disease association by fully connected network

MDAs are predicted using fully connected network, where extracted miRNA-diseases feature are integrated through a fusion mechanism as follow.


(20) $$F=\frac{\;\alpha_{1}F_{1}\;+\;\alpha_{2}F_{2}\;+\;\alpha_{3}F_{3}}{\alpha_{1}\;+\;{\;\alpha }_{2\;}\;+\;\alpha_{3}}$$

where (*F*1, *F*2, *F*3) are feature vector from different module. The coefficient *αᵢ* determines the feature weight. All modules were assigned equal weight *(αᵢ* = *α*), reflecting their equal significance.

After feature extraction, outputs from modules were linearly transformed and fed into a fully connected network to predict MDAs (Yang *et al*. 2023).


(21) y*=sigmoid (W.F+b)

where *W* and *b* represent learnable weight matrix, the model was trained using cross-entropy loss function, and Adam optimizer used to minimize loss function.


(22) $$L=-\frac{1}{N}\;\sum_{i=1}^{N}y_{i}\;\log\;y_{i}+\;(1\;-y_{i})\;\log(1\;-y_{i})$$

where *N* and $y_{i}\;$represent the number of ncRNA-disease association pairs and true labels.

Our proposed HybridGNN synergistically integrates MDMF, GCN and GAT to jointly capture structural graph topology and semantic association patterns in the miRNA-disease network.


(23) $$A\approx {UV}^{T}$$

where $U\;\in \;R^{m\times \iota }$, $V\in R^{d\times \iota }$ Here, *l* is the latent dimension (e.g., *l *= 42). The MDMF loss function combines reconstruction error and regularization. The loss function is explained below:


(24) $$L_{\mathrm{MDMF}}=∥A-\;{UV}^{T}{∥}_{2}^{2}+\lambda (∥U{∥}_{2}^{2}\;+∥{V∥}_{2}^{2}+∥{UU}^{T}-S_{m}{∥}_{2}^{2}+∥{VV}^{T}-S_{d}{∥}_{2}^{2}),$$

where *λ* (e.g., 0.0121) balanced reconstruction and regularization strength.

### 2.8 Training protocol

The heterogeneous graph was constructed using PyG. miRNAs and diseases presented as distinct node types, and miRNA-disease associations presented as relational edges. To ensure rigorous training and evaluation, we employed the RandomLinkSplit function to split edges (Betkier *et al*. 2023). (RandomLinkSplit is a dataset splitting tool specifically designed for the graph link prediction task in the PyTorch Geometric (PyG) library. The core of link prediction is to predict “whether there is an edge between two nodes”, so the core of the process is splitting edges.) The edges were split into 80% training, 10% validation, and 10% test sets, to evaluate the model ability to predict unseen association. Nodes and edges were partitioned differently because due to their distinct roles in the learning. A disjoint-train-ratio parameter of 0.3, assigned 70% of node for training and 30% for supervision. Node splitting controls message passing and embedding learning without evaluating prediction, whereas edge splitting provides supervision for link prediction; thus, they serve complementary but distinct roles.

Regarding the setting of the negative sampling ration, negative edges are dynamically sampled at a 20% (0.2 ratio) of the total negative association during training, to provide sufficient negative supervision and model generalization. During evaluation, the number of negative edges is fixed at 10% (0.1 ratio). The model was trained using Binary Cross-Entropy with Logits Loss, optimized by Adam optimizer (learning rate of 0.0076) and weight decay (1.75 × 10^−5^) (Huang *et al*. 2022). The mini-batch subgraph loaders employed to improve computational efficiency and scalability. Hyperparameter were optimized using optuna tuning with100 trials, selecting the best configuration (e.g hidden size 117) based on the validation AUC (Pham *et al*. 2023, Harun-Or-Roshid *et al*. 2024, Pham *et al*. 2024, Dhanka *et al*. 2026). Early stopping was applied with a patience of 20 epochs to prevent overfitting. The model was trained using the following setup. Loss Function: Binary cross-entropy with logits as shown in Equation (25):


(25) $$L=\;\frac{1}{N}\;\Sigma_{i=1}^{N}[y_{i}\log\left({\hat{y}}_{i}\right)+(1-y_{i})\log+(1-{\hat{y}}_{i})]$$

where $Y_{i}$ is the true binary label, and ${\hat{y}}_{i}$ represents the predicted probability of sample *i*. This equation is particularly suitable for the binary classification task and effectively handle prediction express as logits.

Furthermore, the residual connection was added to prevent vanishing gradients and retain original node features by merging GCN outputs with the transformed outputs as shown in Equation (26).


(26) $$H_{\mathrm{out}}=H_{\mathrm{GCN}}+W_{r}\;H_{\mathrm{in}}$$

where *H*_GCN_ is the GCN output, *H*_in_ is the input feature matrix, $W_{r}$ is a learnable linear projection matrix ensuring stable training and retains key feature. The details of hyperparameters adjustment for the HybridGNN model are also shown in the Table 1.

**Table 1:** Hyperparameter configuration and optimal settings of the HybridGNN model.

Parameter	Search space	Optimal value
Hidden Dimension	[64, 256]	117
Learning Rate	[10⁻⁵, 10⁻²]	0.0076
Dropout	[0.1, 0.5]	0.4493
Number of Heads	[1, 16]	12
Number of Layers	[2, 4]	2
PCA Dimension	{16, 32, 64}	32
Latent Dimension	[16, 64]	42
Output Channels	[8, 64]	22
λreg	[0.01, 0.1]	0.0121
Weight Decay	[10⁻⁵, 10⁻³]	1.75 × 10⁻⁵

### 2.9 Accuracy metrics

To evaluate model performance, multiple metrices employed such as accuracy (ACC), Precision (Pre), Recall (Rec), F1-score, Area Under the Receiver Operating Characteristic Curve (AUC-ROC) and Area Under the Precision-Recall curve (AUPRC). AUC-ROC quantifies the model’s overall discriminative ability by measuring the trade-off between true positives rate (TPR) and against false positive rate (FPR), whereas AUPRC emphasizes precision-recall behavior, and is particularly informative for imbalanced datasets. In Addition, ACC, Pre, Rec, and F1-score were computed to provide a balanced assessment of classification performance. All evaluation metrics, were computed in Equation (27) (Liu *et al*. 2019, Liu *et al*. 2024a, Meng *et al*. 2024, Shao *et al*. 2025, Xing *et al*. 2025, Huang *et al*. 2025).


(27) {ACC=TP+TNTP+TN+FP+FNRec=TPTP+FNPre=TPTP+FP F1=2.precision.recallprecision+recall

where TP represents the number of correctly identified positive samples, TN denotes the number of correctly classified negative samples, FP refers to the number of falsely predicted positive samples, and FN indicates the number of misclassified negative samples.

## 3 Results

### 3.1 HybridGNN model results

**Figure 2:**
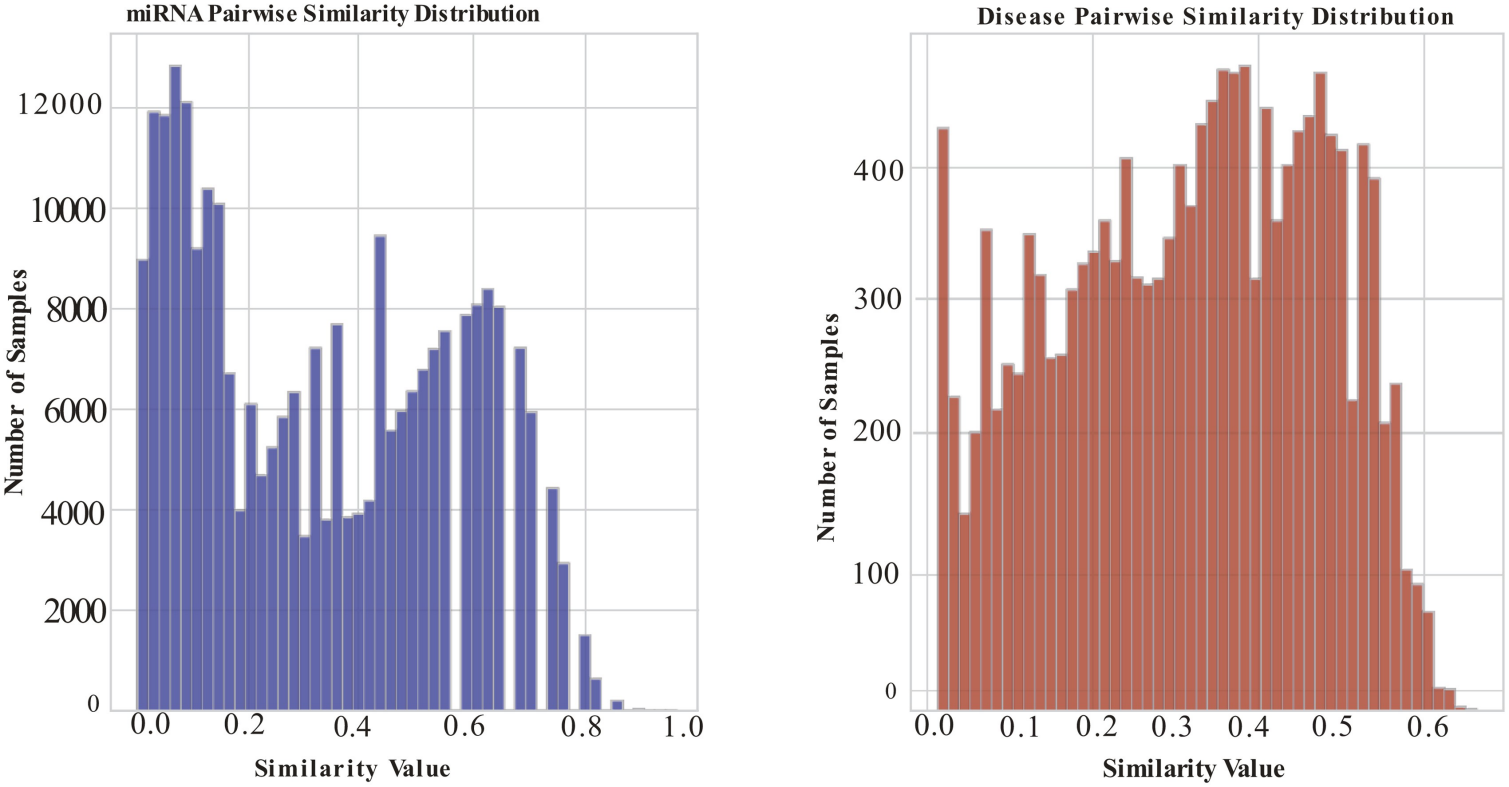
Distribution of pairwise similarity values between miRNAs and diseases matrices. A pair correlation that is weaker and stronger indicated by the values closer to 0 and 1.

The dataset was obtained from HMDD v4.0 database, The dataset captures pairwise similarities between miRNAs and diseases. Analyzing their distribution reveals functional relationships, offering valuable insights into biological interactions. We calculated the similarity values for miRNAs and disease, explored the distribution in Fig. 2. The verticals axis represents the number of samples within the specific similarity ranges, while the horizontal axis explores the range of similarity values of miRNAs and disease. The miRNAs-miRNAs and disease-disease similarity scores mostly range between 0 and 0.8. This similarity results were higher compared with the similarity distribution reported in previous study. For instance, the SAGESDA model reported low snoRNA-snoRNA and disease-disease similarity values with most score concentrated between 0.0 and 0.2 (Momanyi *et al*. 2024b). The higher similarity score in the current study can be attributed to the fact that we considered the average of three similarity scores measures for miRNA–miRNA pairs and average of two similarity measures for disease-disease pairs, which more effectively capture the underlying biological relationships.

Building upon this foundation, we developed HybridGNN, model for predicting MDAs by integrating three heterogeneous networks and a dot-product classifier. The model, trained with mini-batch subgraph loaders, ensures scalability, efficiency, and reduces over-smoothing. HybridGNN employs multi-head attention in GAT for informative neighbor aggregation, with a penalty term (*λ*) to avoid overfitting. Although attention increases time complexity, it improves edge-specific weight learning. Shared weights across networks provide richer node embeddings. Cosine similarity in the dot product quantifies miRNA-disease alignment. HybridGNN can compute embeddings for new miRNAs or diseases without retraining, offering enhanced scalability, accuracy, and adaptability.

**Figure 3:**
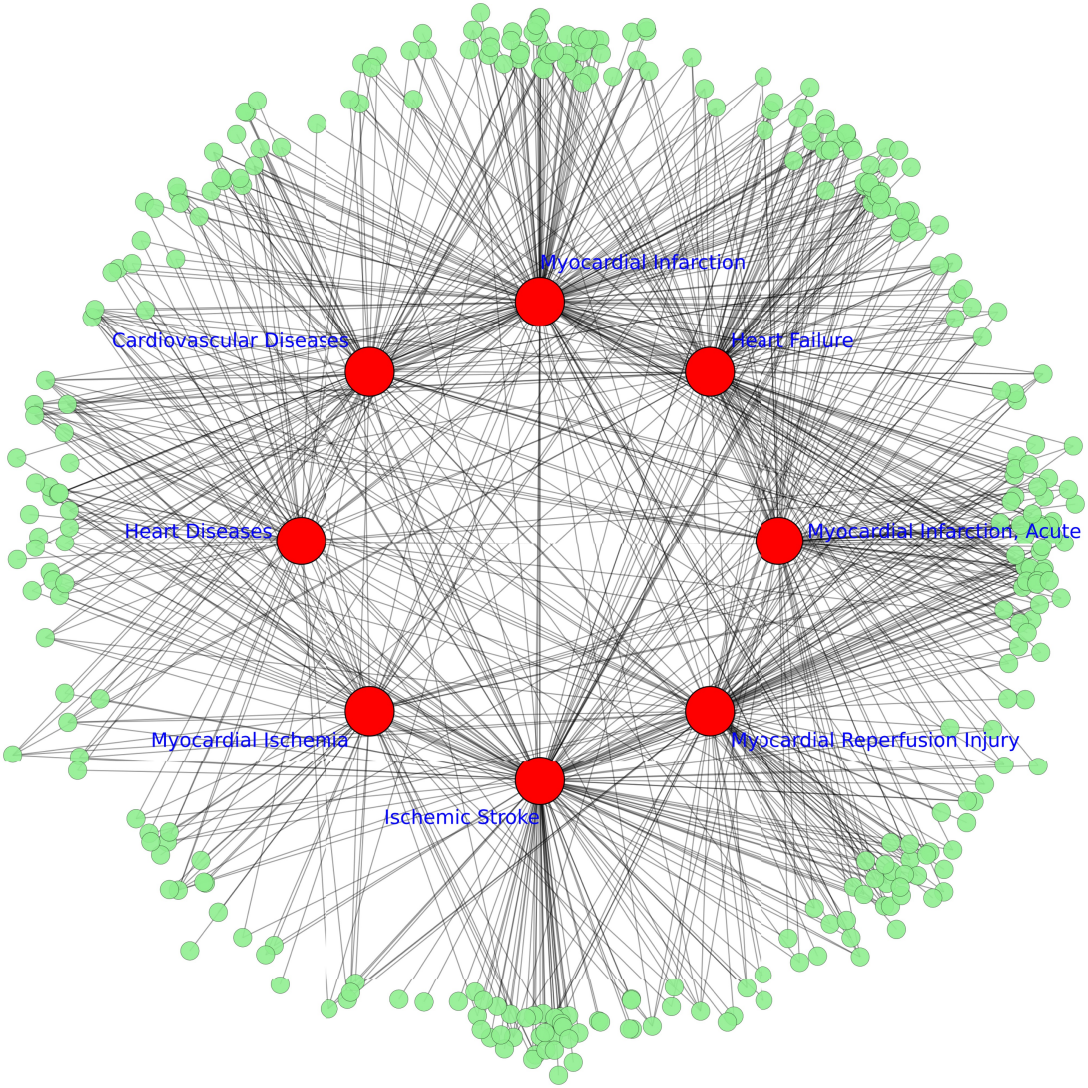
Illustration of the heterogeneous graph network showing two node types miRNA (green) and diseases (red) connected by undirected links that enable bidirectional message propagation and effective feature aggregation in HybridGNN model.

To visualize miRNA-disease associations, we selected the subnetwork of heart-related diseases, because these diseases exhibited highest number of miRNA association (Fig. 3). The highest miRNA frequencies refer to the number of known and validated miRNA-disease associations recorded for each disease in the integrated heterogeneous network. The bipartite subgraph included 820 pairs linking 324 miRNAs (green) and 8 diseases (red). Central clustering of myocardial infarction and ischemic stroke indicated strong connectivity, while peripheral miRNAs showed disease-specific patterns.

**Figure 4:**
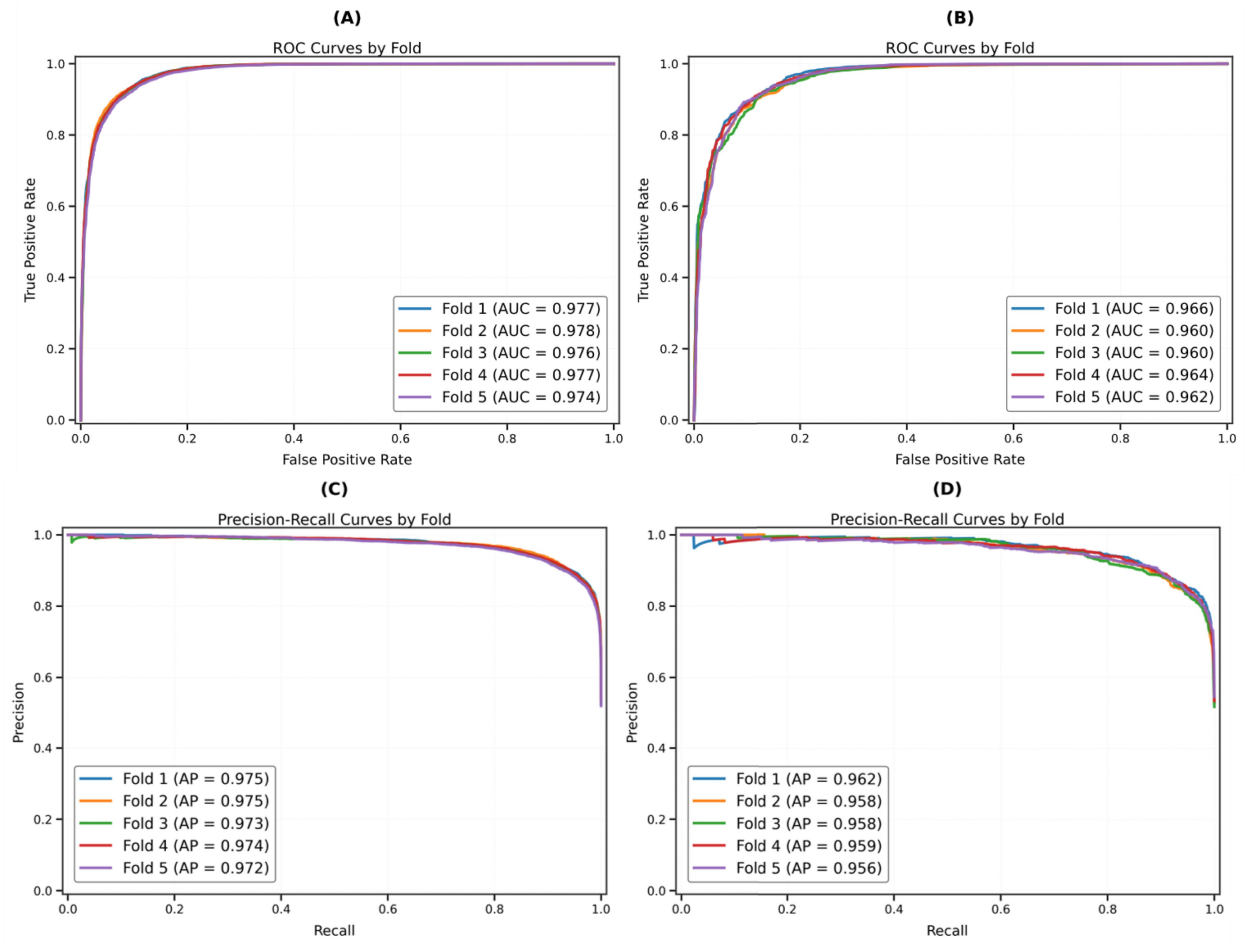
Evaluation results of the HybridGNN model on proposed dataset and IMCMDA dataset: (A) ROC curve on the proposed dataset, (B) ROC curve on the IMCMDA dataset (C) PR curve on proposed dataset and (D) represent PR curve on the IMCDMA dataset.

Based on constructed miRNA-disease heterogenous networks, the model performance was evaluated by comparing the predicted association scores with ground-truth data from the evaluation dataset, encompassing both positive and negative associations. To ensure robustness and stability five-fold cross-validation (FF-CV) was employed on proposed dataset. As shown in Fig. 4A and C, HybridGNN achieves AUC values above 0.97 and Average precision values around 0.97 across all folds on proposed dataset, demonstrating excellent discriminative ability, stable performance, and strong capability in identifying true miRNA-disease associations, even under class-imbalance conditions. The generalization ability of HybridGNN was evaluated on independent IMCMDA dataset. As shown in Fig. 4B and D, model maintains strong predictive performance, achieving AUC values around 0.96 and AP values above 0.95 across five folds. The ROC and PR curves remain smooth and stable, indicating consistent predictive behavior across different data splits. Compared with previously reported methods on the IMCMDA dataset, which achieved an AUC of 0.8845 and an AUPR of 0.8832, HybridGNN demonstrates a substantial improvement, achieving an AUC of 0.96, an accuracy of 0.89, and an F1-score of 0.89. These results underscore the robustness and effectiveness of the HybridGNN framework for miRNA-disease association prediction.

In this study, the cold disease setting refers to an evaluation scenario in which all associations related to certain diseases are completely excluded from the training set and used only for testing. This setting evaluates the model’s ability to predict miRNA associations for previously unseen dis eases. Similarly, the cold miRNA setting refers to a scenario where all associations corresponding to specific miRNAs are removed from the training data and reserved for testing. This strategy assesses the model’s capability to generalize to novel miRNAs without prior association information as shown in Table 2.

**Table 2:** HybridGNN model performance on an IMCMDA dataset.

Split mode	AUC (mean ± std)	AUPR (mean ± std)	ACC (mean ± std)	F1 (mean ± std)
random	0.9625 ± 0.0022	0.9586 ± 0.0018	0.8928 ± 0.0024	0.8953 ± 0.0021
cold_disease	0.9437 ± 0.0092	0.9352 ± 0.0072	0.8613 ± 0.0235	0.8684 ± 0.0180
cold_mirna	0.9583 ± 0.0042	0.9552 ± 0.0069	0.8866 ± 0.0077	0.8894 ± 0.0071

### 3.2 Ablation study

To investigate the contribution of each architectural component and feature type, we conducted an ablation study of HybridGNN model. The performance was evaluated using AUC evaluation. As shown in Table 3, the full HybridGNN model, which integrates a hybrid GCN-GAT architecture, GIP and MDMF features, similarity-based edges, and residual connections, achieves the best overall performance with AUCs of 0.9765. Removing either GAT and GCN leads to noticeable performance degradation. The GAT-only variant shows a slight drop in AUC (0.9708) indicating that attention alone is insufficient to capture all topological patterns. Similarly, the GCN-only variant exhibits lower Random AUC (0.9386), demonstrating that combining GCN and GAT enables more expressive node representations. MDMF features are also important, as their removal leads to a noticeable drop in performance as evidenced by decreased in AUC 0.9285. This indicates that MDMF features significantly enhance association prediction. The baseline GCN and GAT models without similarity edges and MDMF features achieve the lowest AUC scores across all evaluation settings. In particular, Baseline-GAT yields an AUC of only 0.8951, confirming that the proposed enhancements feature fusion, similarity edges, and hybrid architecture are essential for robust performance. Overall, the ablation results demonstrate that each component of the proposed HybridGNN contributes positively to performance, with the hybrid GCN–GAT architecture and MDMF features playing particularly critical roles in improving generalization under different evaluation setting.

**Table 3:** Ablation study component contribution analysis (AUC).

Variant	Model architecture	GIP Features	MDMF features	Similarity edges	Residual connections	AUC
FULL (HybridGNN)	GCN + GAT Hybrid	✓	✓	✓	✓	0.9765
GAT-Only	GAT Only	✓	✓	✓	✓	0.9708
Only-MDMF	GCN + GAT Hybrid	✗	✓	✓	✓	0.9705
GCN-Only	GCN Only	✓	✓	✓	✓	0.9386
No-MDMF	GCN + GAT Hybrid	✓	✗	✓	✓	0.9285
Baseline-GCN	GCN Only	✓	✗	✗	✓	0.9254
Baseline-GAT	GAT Only	✓	✗	✗	✓	0.8951

### 3.3 Comparative performance evaluation with previous methods

**Figure 5:**
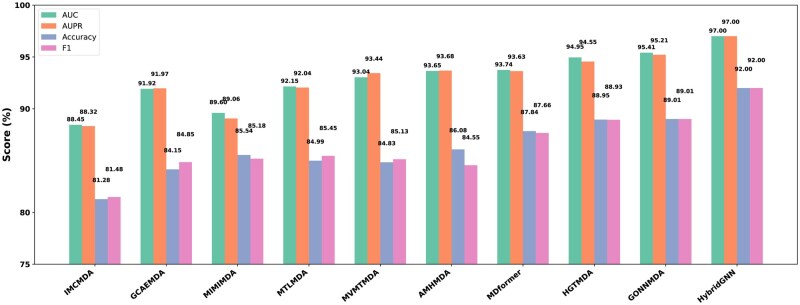
Comparative analysis of the different models with HybridGNN model based on the AUC, AUPR, Accuracy and F1.

We compare HybridGNN model with several state-of-the-art methods, in addition to quantitative metrics, receiver operating characteristic (ROC) and precision-recall (PR) curves are plotted to provide a comprehensive and visual assessment of predictive performance. The comparative IMCMDA methods (Chen *et al*. 2018a) employs neural inductive matrix completion framework to infer potential miRNAs–disease association (MDAs). GCAEMDA (Li *et al*. 2021a) utilized graph convolutional autoencoder to model association patterns and MINIMDA (Lou *et al*. 2022) constructs a similarity network by integrating multi-source miRNA information and capture high-order domain features followed by a multilayer perceptron for associations prediction. MTLMDA (He *et al*. 2023) applies a multi-task learning strategy to predict potential MDAs. While VMTMDA (Huang *et al*. 2021) model employed the multi-view, multi-task approach, to predict MDAs. AMHMDA (Ning *et al*. 2023) incorporates graph convolutional networks with attention mechanisms to predict MDAs. furthermore, MDformer (Dong *et al*. 2023) model leverages a transformer-based architecture and combined with meta-path information to predict the MDAs. HGTMDA (Lu *et al*. 2024) integrates graph convolutional attention with a transformer encoder to predict MDAs scores. Finally, GONNMDA (Zeng *et al*. 2025) applies graph neural network to the heterogeneous biological molecular graph, for effective MDAs. In Fig. 5, the comparison highlights that HybridGNN model is superior from all models. These complementary metrics allowed for a more holistic evaluation of the model’s predictive performance. Its advantage comes from dynamic embedding updates and advanced feature learning. However, HybridGNN integrates MDMF with PCA to fuse heterogeneous features and remove redundancy. GNN layers with GAT assign dynamic node-specific weights for refined feature processing. The findings indicate that the proposed HybridGNN approach consistently outperforms from existing methods, providing compelling evidence of its robustness and superiority across multiple evaluation criteria.

### 3.4 Impact of data split strategies and stratified performance analysis

To evaluate data leakage and strengthen confidence in model performance, we employed three distinct data-split strategies. These includes a random split using 20% of miRNA-disease pairs, a 15% cold-disease split in which disease entities in the test set do not appear during training, and a 15% cold-miRNA split in which miRNA entities in test set are unseen during training. The results of these splitting strategies are presented in Table 4 based on 5-fold cross-validation performance metrics.

**Table 4:** Evaluation metrics of HybridGNN across random and cold-start setting.

Metric	Random split	Cold-disease split	Cold-miRNA split	Performance pattern
Mean AUC-ROC (±SD)	0.9765 ± 0.0006	0.9365 ± 0.0054	0.9678 ± 0.0009	Random > Cold-miRNA > Cold-Disease
Mean PR-AUC (±SD)	0.9734 ± 0.0008	0.9397 ± 0.0160	0.9675 ± 0.0007	Consistent with AUC trends
Mean Accuracy (±SD)	0.9186 ± 0.0028	0.5756 ± 0.0279	0.9034 ± 0.0024	Cold-Disease shows classification difficulty
Mean Precision (±SD)	0.9013 ± 0.0057	0.5424 ± 0.0170	0.8966 ± 0.0097	Cold-Disease: low precision, high recall
Mean Recall (±SD)	0.9403 ± 0.0056	0.9809 ± 0.0079	0.9123 ± 0.0106	Cold-Disease: recall-oriented predictions
Mean F1-Score (±SD)	0.9204 ± 0.0026	0.6983 ± 0.0123	0.9042 ± 0.0022	Balanced performance in non-cold scenarios
Performance Stability	Highest (lowest std)	Moderate	High	Random split most consistent

Moreover, we conducted a stratification analysis based on seven disease categories to assess the generalization ability of Hy-bridGNN and its performance analysis. Table 5 shows category dependent performance variations across evaluation settings. While random split yield uniformly high performance, cold-disease split more severely affect genetic/syndrome, neurological disorder and other diseases. Fibrotic diseases generalize well under cold-start conditions, whereas Inflammatory and Cardiovascular diseases remain relatively stable. Performance under cold-miRNA splits is consistently high, indicating greater robustness to unseen miRNAs than to unseen diseases.

**Table 5:** HybridGNN performance across disease categories under different split.

Disease category	Random split	Cold-disease split	Cold-miRNA split	Performance pattern	Biological interpretation
Cancer	0.9780	0.9661	0.9770	Consistently high across all splits	Well-characterized associations with clear miRNA patterns; extensive training data enables robust generalization
Fibrotic	0.9643	0.9725	0.9798	Improves in cold scenarios	Distinct, focused miRNA involvement allows easier pattern recognition even with novel entities
Neurological	0.9791	0.9140	0.9778	Cold-disease vulnerable	Complex, multi-system involvement; associations less consistent across different neurological conditions
Genetic/Syndrome	0.9747	0.8904	0.9576	Most cold-disease sensitive	Rare conditions with limited training examples; heterogeneous molecular mechanisms
Inflammatory	0.9662	0.9246	0.9693	Stable across splits	Moderately characterized with consistent immune-related miRNA patterns
Cardiovascular	0.9762	0.9207	0.9730	Moderate cold-drop	Well-studied but with diverse pathological mechanisms affecting generalization
Other	0.9745	0.9117	0.9633	Significant cold-disease drop	Heterogeneous category; lack of clear unifying patterns hampers novel disease prediction

### 3.5 Case study

To validate the predictive ability of HybridGNN model in the prediction of MDAs, we have conducted case study on five diseases: Myocardial Infarction (MI), Ischemic Stroke (IS), Lung Neoplasms (LN), Breast Neoplasm (BN) and Fibrosis (F). These diseases are widely reported in the literature to be strongly regulated by miRNAs, making them suitable benchmarks for miRNA-disease association prediction studies, Additionally, these diseases contain a relatively large number of experimentally validated miRNA associations in the Human microRNA Disease Database, ensuring sufficient data for meaningful case study validation and reducing bias caused by extremely sparse disease nodes and selected diseases cover cardiovascular disorders. This diversity allows comprehensive evaluation of HybridGNN across different biological mechanisms and disease categories rather than focusing on a single disease type (Ha 2011, Çakmak and Demir 2020). Table 6 presents the top 10 miRNAs with the highest predicted scores for each disease according our model prediction (Groenewegen *et al*. 2020). Previous studies have also confirmed the involvement of miRNAs in disease development. For instance, the first two MI-associated miRNA identified in the Table 6, homosapiens (hsa-miR-29a) and hsa-miR-29b, were downregulated in response to myocardial infarction (van Rooij *et al*. 2008). While has-miR-195 was up-regulated during cardiac hypertrophy (Long *et al*. 2012). Similarly, hsa-miR-223 shown downregulated in acute myocardial infarction (Rangrez *et al*. 2013).

**Table 6:** HybridGNN model identified the top 10 most significant miRNA-disease associations.

MI-miRNAs	Evidence	IS-miRNAs	Evidence	LN-miRNAs	Evidence	BN-miRNAs	Evidence	F-miRNAs	Evidence
hsa-mir-29a	Confirmed (Shao et al. 2025)	hsa-mir-21	Confirmed (Xing et al. 2025)	hsa-mir-23a	Confirmed (Ning et al. 2023)	hsa-mir-146a	Confirmed (Groenewegen et al. 2020)	hsa-mir-29b	Confirmed (Zhang et al. 2023)
hsa-mir-29b	Confirmed (Shao et al. 2025)	hsa-mir-29b	Confirmed (Huang et al. 2021)	hsa-mir-29b	Confirmed (Huang et al. 2021)	hsa-mir-23a	Confirmed (van Rooij et al. 2008)	hsa-mir-150	Confirmed (Liang et al. 2024)
hsa-mir-195	Confirmed (Long et al. 2012)	hsa-mir-126	Confirmed (Ha 2025)	hsa-mir-27b	Confirmed (Dong et al. 2023)	hsa-mir-24	Confirmed	hsa-mir-375	Confirmed (Wang et al. 2021)
hsa-mir-143	Confirmed	hsa-mir-34a	Confirmed (Li et al. 2025)	hsa-mir-22	Confirmed (Lu et al. 2024)	hsa-mir-205	Confirmed (Rangrez et al. 2013)	hsa-mir-29	Confirmed (Zhu et al. 2020)
hsa-mir-223	Confirmed (Shao et al. 2025)	hsa-mir-125a	Confirmed (Lou et al. 2022)	hsa-mir-375	Confirmed (Zeng et al. 2025)	hsa-mir-23b	Confirmed (Zhan et al. 2023)	hsa-mir-451	Confirmed (Pastuszak-Lewandoska et al. 2016)
hsa-mir-144	Confirmed	hsa-mir-206	Confirmed (He et al. 2023)	hsa-mir-126	Confirmed	hsa-mir-192	Confirmed	hsa-mir-200a	Confirmed
hsa-mir-15b	Confirmed	hsa-mir-146b	Confirmed	Has-mir-101	Confirmed	hsa-mir-210	Confirmed	hsa-mir-122	Confirmed
hsa-mir-214	Confirmed	hsa-mir-204	Confirmed	hsa-mir-641	Confirmed	hsa-mir-138	Confirmed	hsa-mir-29b-1	Confirmed
hsa-mir-218	Confirmed	hsa-mir-9	Confirmed	hsa-mir-340	Confirmed	hsa-mir-146b	Confirmed	hsa-mir-374a	Confirmed
hsa-mir-7	Confirmed	hsa-mir-19a	Confirmed	hsa-mir-152	Confirmed	hsa-mir-556	Confirmed	hsa-mir-1202	Confirmed

**MI = Myocardial Infarction, IS = ischemic stroke, LN = Lung Neoplasms, BN = Breast Neoplasm, F = Fibrosis ** confirmed indicates that miRNA is associated with disease as predicted by model.

Among IS-related miRNAs, hsa-mir-21 is upregulated and plays regulatory role highlighting the importance of miRNAs in Ischemic stroke (Zhan *et al*. 2023). Hsa-mir-126 has been proposed as a potential biomarker for ischemic stroke (Zhang *et al*. 2023). Hsa-mir-34a was reported up-regulated in blood samples of IS patients (Liang *et al*. 2024). The hsa-miR-125 family has been implicated in the pathogenesis of ischemic stroke (Wang *et al*. 2021). Hsa-miR-206, has identified a potential diagnostic marker for ischemic stroke (Zhu *et al*. 2020).

For LN-miRNAs, hsa-miR-29b is downregulated in NSCLC tissues, and its reduced expression correlates with lymphatic metastasis (Pastuszak-Lewandoska *et al*. 2016). The overexpression of hsa-miR-23a/27a/24–2 cluster promotes non-small cell lung cancer progression suggesting its role as an oncogenic regulatory unit in tumor growth (Fan *et al*. 2022). Our study revealed that hsa-miR-27b is associated with lung neoplasm, and participates in angiogenesis. Highlighting its potential therapeutic target in cancer (Ding *et al*. 2017). The involvement of hsa-mir-22 in lung neoplasms is consistent with prior studies, suggesting its potential anti-angiogenic therapeutic target in non-small cell lung cancer (Gu *et al*. 2021). Notably, hsa-miR-375 is associated with lung neoplasm, and may act as an oncogene in certain types of cancer, as reported in previous studies (Nishikawa *et al*. 2011).

Among BN-miRNAs, hsa-miR-146a identified potential regulator linked to lung neoplasm consistent with its reported role in breast cancer proliferation (Chen *et al*. 2020). The hsa-miR-23a is associated with lung cancer, aligning with previous evidence that its down-regulation contributes to breast cancer (Chen *et al*. 2017). A strong association between hsa-miR-205 and breast cancer was predicted, while the previous results showing its overexpression in breast cancer (Xiao *et al*. 2019). Our result demonstrates that hsa-miR-23b act as an oncogene in breast cancer, and its predicted association is consistent with existing experimental evidence (Hannafon *et al*. 2019).

For F-miRNAs, hsa-miR-29b showed a strong association with fibrosis, existing literature confirmed that miR-29a and miR-29b are linked to renal fibrosis (Wang *et al*. 2012). The detection of the hsa-miR-150 in the current case study underscore its potential role in modulation of liver fibrosis (Tian *et al*. 2025). Consistently, miR-29 is significantly downregulated in human fibrotic disorders of multiple organs (Cushing *et al*. 2015). Our analysis reveals that miR-451 has a potential role in fibrosis aligning with previous studies that this miRNA identified as a promising biomarker for cardiac fibrosis (Jin 2021).

## 4 Discussion

Emerging evidence suggests that many miRNA genes are located within specific genomic regions associated with various diseases, indicating their key regulatory role in disease progression (Zhong *et al*. 2024, Liu *et al*. 2025). Consequently, accurate prediction of miRNA-disease associations (MDAs) has become a key area of research. Although, numerous machine learning and deep learning models have achieved promising results, many still face challenges related to interpretability, scalability, and computational cost. Therefore, developing more efficient and interpretable computational frameworks remains essential for advancing disease diagnosis, prognosis, and therapeutic discovery (Sheng *et al*. 2024). In this study, we employed HybridGNN a heterogeneous network-based model for predicting potential MDAs. Three types of miRNA similarities and two types of disease similarities were integrated to construct unified similarities metrices which served as adjacency representations of miRNA-disease network. The model incorporated MDMF coupled with principal component analysis (PCA), GCN, and GAT to capture diverse information from network. A transition layer fused these multi-source features into a unified dimension and a fully connected neural network predicted the final association scores between miRNA-disease pairs.

Ablation analysis revealed that no single architectural configuration consistently dominates across all evaluation scenarios, emphasizing scenario-dependent optimality. While the GAT-only variant achieved performance comparable to the full model under random splits, GCN demonstrated stronger generalization in cold-disease settings, likely due to its simpler aggregation mechanism. In contrast, optimal performance in miRNA prediction required the full HybridGNN architecture, confirming the synergistic contribution of GCN, GAT, and feature fusion components. Furthermore, MDMF features were shown to capture distinct yet complementary similarity information, reinforcing the benefit of multi-source feature integration.

In recent years, multiple deep learning-based models have been proposed to enhance (MDA) prediction. Notably, IMCMDA (Chen *et al*. 2018a), employed an inductive matrix completion to integrates functional similarity, semantic similarity, achieving an AUC of 0.8034. GCAEMDA (Li *et al*. 2021) used graph convolutional autoencoder to learn embeddings from heterogeneous similarity network reporting an AUC of 0.9415. MINIMDA (Lou *et al*. 2022) incorporated a high-order neighborhood information to improve representation learning achieving an AUC of 0.9. MTLMDA (He *et al*. 2023) introduced a multi task GCN based framework, while MVMTMDA (Huang *et al*. 2021) and AHMDA (Ning *et al*. 2023) further explored multitask and attention-based learning strategies for MDA prediction.

To compared with these methods, HybridGNN consistently achieved superior AUC AUPR, demonstrating its effectiveness in modelling complex biological associations. Nonetheless, the model presents limitation including increasing computational complexity due to multi-head attention mechanism, reliance on local neighborhood information, and evaluation on a single dataset. Future work will focus on optimizing computational efficiency, incorporating gradual component analysis, validating across multiple datasets and integrating additional biological features such as expression or pathway data. Extending HybridGNN framework to related tasks including protein-disease and drug-target association prediction, may further enhance its generalizability and predictive power.

## 5 Conclusion

In this study, we proposed a HybridGNN that integrates features from three powerful methods Matrix Decomposition with Matrix Factorization (MDMF), Graph Convolutional Network (GCN), and Graph Attention Network (GAT). This integrated framework is designed to significantly enhance the prediction of miRNA-disease associations. The model first employs a multi-similarity network to extract features, followed by three specialized feature extraction modules. A fully connected network is then used to predict miRNA-disease association scores, uncovering previously unknown associations. Owing to this multi-step process, the model achieved a higher AUC score compared to existing approaches, indicating superior predictive performance. Furthermore, we conducted case studies on heart-related diseases such as Myocardial Infarction (MI) and Ischemic Stroke (IS), Lung Neoplasms, Breast Neoplasm, Fibrosis the model identified miRNAs association, confirming its potential for predicting meaningful associations that could aid in future clinical diagnostics. Overall, the development of HybridGNN model cover a critical gap in the prediction of miRNA-disease associations and paves the way for future research, ultimately contributing to advancements in precision medicine and improved healthcare outcomes.

## Data Availability

The dataset of miRNA-disease associations in this article can be obtained from Human microRNA disease database (HMDD v4.0).
